# Clinical spectrum and mechanistic insights into cardiotoxicity from cancer therapies

**DOI:** 10.1007/s11239-026-03336-x

**Published:** 2026-06-02

**Authors:** Richard C. Becker

**Affiliations:** University of Cincinnati College of Medicine, 231 Albert Sabin Way, Cincinnati, OH 45267 USA

**Keywords:** Cardiotoxicity, Cancer Therapeutics, Preventive Strategies

## Abstract

Cardiotoxicity is a major concern in oncology, influencing both patient safety and therapeutic decision-making. Part 1 of a dedicated 3-part series provides a comprehensive overview of the fundamentals of cardiotoxicity associated with cancer therapeutics, beginning with a definition of cardiotoxicity and classifying its mechanisms into direct myocardial injury, indirect cardiovascular effects, and a combination of both. Clinical manifestations are examined across the spectrum of presentations, from asymptomatic cardiac biomarker elevations to overt heart failure, alongside current detection strategies including imaging and patient-reported outcomes. Timing and incidence patterns are analyzed to highlight early versus delayed toxicity and their implications for surveillance strategies used in clinical practice. Guideline-directed therapies are summarized and suggestions for drug development that concomitantly begins to frame cardiotoxic effects are introduced. Lastly, the impact of cardiotoxicity on cancer treatment decisions, including dose modifications, therapy discontinuation, multidisciplinary management, is considered to establish a clear context for optimal patient care.

## Drug associated cardiotoxicity

Cardiotoxicity is generally defined as the direct or indirect toxic effect of a drug, chemical, or therapeutic intervention on the heart, resulting in structural or functional cardiac injury. In the context of cancer therapeutics, the American Heart Association (AHA) defines cardiotoxicity as an absolute decline in left ventricular ejection fraction (LVEF) of ≥ 5% to < 55% with heart failure symptoms, or an asymptomatic decrease in LVEF ≥ 10% to < 55% during treatment [1, 2].

**Fig. 1:**
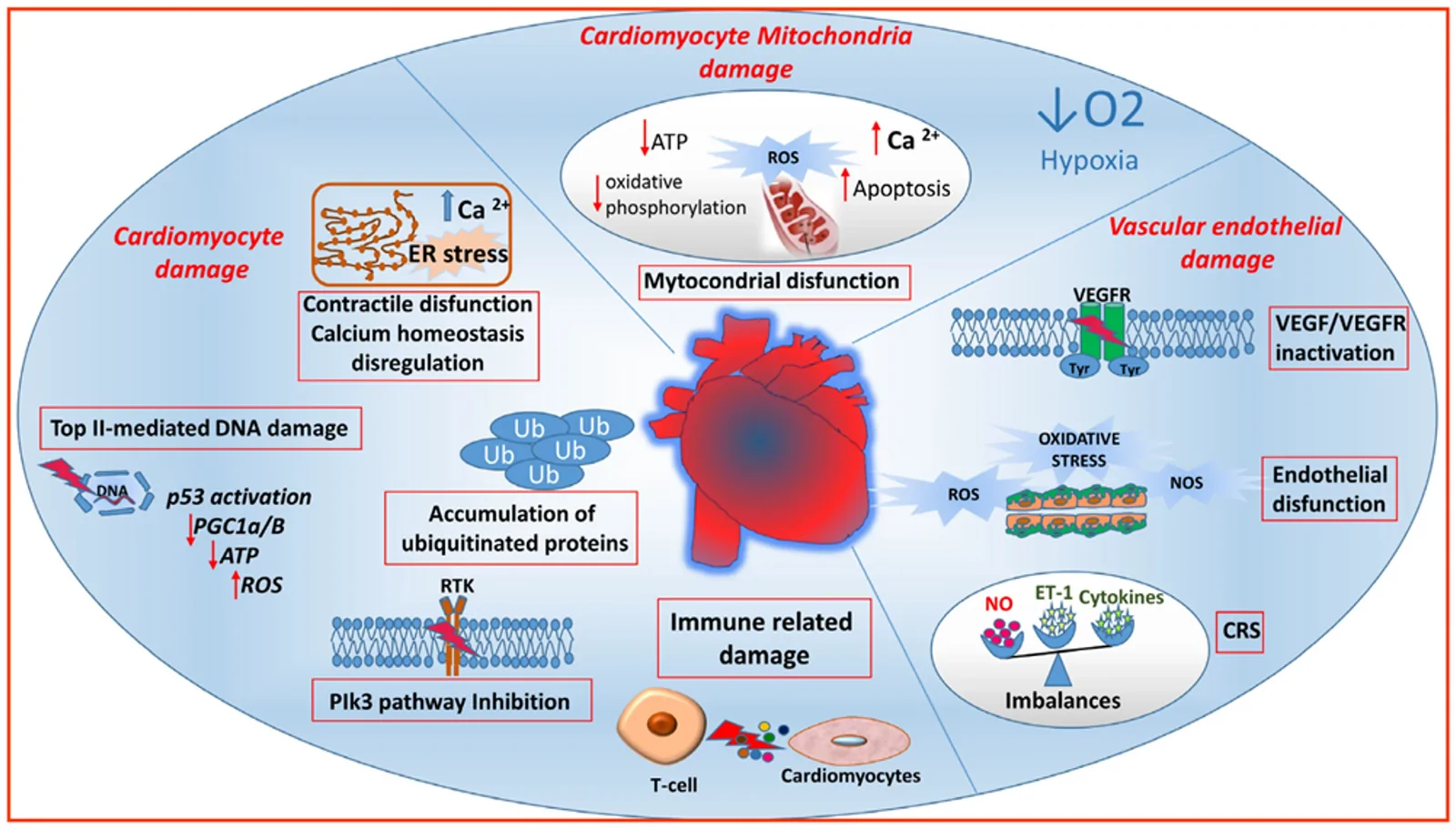
Molecular mechanisms of anticancer drug-mediated cardio-vascular toxicity. Chemotherapeutic agents and targeted treatments can induce cardiovascular toxicity by affecting both endothelial cells and cardiomyocytes. In vascular endothelial cells, the inhibition of the VEGF pathway can result in hypertension, altered vascular permeability, bleeding, and thrombosis. In endothelial cells, oxygen reactive species (ROS) can disrupt the equilibrium between vasoconstricting agents, including endothelin (ET-1), and vasodilating agents such as nitric oxide (NO) produced by nitric oxide synthase (NOS). In the cytokine release syndrome (CRS), vasoactive soluble factors cause vasodilation and hypotension. The impact of antineoplastic drugs on cardiomyocytes may interest the mitochondria and could be linked to hypoxia resulting from vascular damage or to a direct influence of the drugs on the mitochondria, especially on the respiratory chain. This can result in ATP depletion, heightened ROS, calcium ion release (Ca^2+^), and apoptosis. Additionally, drugs can induce damage to cardiomyocytes by causing endoplasmic reticulum (ER) stress, DNA damage due to topoisomerase 2β (Top II) inhibition, accumulation of ubiquitinated (Ub) proteins, and interference with intracellular pathways, including PI3K, through receptor tyrosine kinase (RTK) inhibition. Top II caused cardiomyocyte death by p53 activation leading to ROS generation and reduction of ATP and peroxisome proliferator–activated receptor-γ coactivator 1-α and 1-β (PGC1α/β). Furthermore, immune checkpoint inhibitors can stimulate T-lymphocytes to recognize self-antigens, leading to myocarditis. From: Barachini S, Buda G, Petrini I. Cardiovascular Toxicity of Antineoplastic treatments in Hematological Diseases: Focus on Molecular Mechanisms to Improve Therapeutic Management. J. Clin. Med. 2024, 13(6), 1574

The term *drug-associated cardiotoxicity* is increasingly favored over simply cardiotoxicity in the context of cancer treatment because it provides greater specificity and clinical clarity. While cardiotoxicity broadly refers to any toxic effect on the heart, it does not indicate causality. By contrast, drug-associated cardiotoxicity explicitly attributes cardiac damage to pharmacological agents or other therapeutics used in the management of patients with cancer (Fig. 1). Specifying that toxicity is drug-associated, clinicians and researchers can better assess causality, tailor monitoring strategies, and design interventions that are specific to the pharmacologic agent involved (reviewed in Nagy et al. [3].

The definition of cardiotoxicity has broadened in recent years to encompass not only ventricular dysfunction but also a spectrum of cardiovascular toxicities, including arrhythmias, myocarditis, pericardial disease, hypertension, myocardial ischemia, and thromboembolic events, as recognized by the American Society of Echocardiography (ASE), the European Society of Cardiovascular Imaging (ESCI), and the European Society of Cardiology (ESC) [4]. Cardiotoxicity may be acute, subacute, or chronic, and can manifest itself as subclinical myocardial injury detectable by biomarkers (e.g., high sensitivity troponin) or imaging, such as decreasing global longitudinal strain preceding a change in left ventricular ejection fraction or overt clinical heart failure [5] (Table 1).

**Table 1:** Broadened Definition of Cardiotoxicity

Aspect	Details
Broadened Definition	Cardiotoxicity now includes not only ventricular dysfunction but also a spectrum of cardiovascular toxicities: arrhythmias, myocarditis, pericardial disease, hypertension, myocardial ischemia, thromboembolic events.
Recognizing Organizations	American Society of Echocardiography (ASE), European Society of Cardiovascular Imaging (ESCI), European Society of Cardiology (ESC).
Temporal Classification	Acute, Subacute, Chronic
Manifestations	Subclinical myocardial injury detectable by biomarkers (e.g., high-sensitivity troponin), Imaging changes (e.g., decreasing global longitudinal strain preceding LVEF change), Overt clinical heart failure
References	[4, 5]

*Direct cardiotoxic effects* refer to the immediate and primary actions of a drug on cardiac cells, particularly cardiomyocytes. These effects often involve structural damage such as cell death, mitochondrial dysfunction, and oxidative stress, are typically dose-dependent and may be irreversible, especially when the damage involves loss of viable cardiac muscle cells.

Direct clinical effects of cardiotoxicity from cancer therapy include left ventricular dysfunction, heart failure, arrhythmias (atrial fibrillation, ventricular tachycardia), myocarditis, pericarditis, coronary artery disease, and sudden cardiac death [6]. In addition, these effects are agent specific. For example, anthracyclines and Human Epidermal Growth Factor Receptor.

(HER)-2-directed therapies are most associated with heart failure, while alkylating agents, taxanes, and antimetabolites can cause tachyarrhythmias, myocardial ischemia, and pericardial disease [7].

*Indirect cardiotoxic effects*, on the other hand, arise from the drug’s impact on non-cardiomyocyte components of the cardiovascular system or through systemic pathways. These may include endothelial cell dysfunction, immune-mediated inflammation, or alterations in vascular tone and integrity. These effects may manifest as arrhythmia, hypertension, or ischemia, and are often influenced by interactions between cardiac fibroblasts, macrophages, and vascular smooth muscle cells [8] (Table 2).

**Table 2:** Direct vs. Indirect Cardiotoxic Effects

Dimension	Direct Cardiotoxic Effects	Indirect Cardiotoxic Effects
Primary Target	Cardiomyocytes (and their organelles such as mitochondria).	Non-cardiomyocyte components of the CV system: endothelium, immune cells, vascular smooth muscle, fibroblasts.
Core Mechanisms	Structural cellular injury: myocyte death, mitochondrial dysfunction, oxidative stress, DNA damage; may involve topoisomerase IIβ inhibition (e.g., anthracyclines).	Endothelial dysfunction, immune-mediated inflammation (e.g., myocarditis from ICI), altered vascular tone/integrity, microvascular spasm, fibroblast–macrophage crosstalk.
Dose Relationship	Often dose-dependent (e.g., cumulative anthracycline dose).	May be dose-independent or idiosyncratic, frequently immune- or stress-mediated.
Reversibility	Often less reversible, especially with loss of viable myocytes; chronic remodeling can ensue.	Frequently potentially reversible if the precipitating mechanism is controlled (e.g., inflammation, vasospasm, BP).
Typical Clinical Manifestations	Left ventricular dysfunction, heart failure, arrhythmias (AF, VT), myocarditis, pericarditis, coronary artery disease, sudden cardiac death.	Arrhythmias, hypertension, ischemia (microvascular or vasospastic), endothelial injury–related events; hemodynamic lability.
Agent Associations	Anthracyclines and HER2-directed therapies → heart failure. Alkylators, taxanes, antimetabolites → tachyarrhythmias, ischemia, pericardial disease.	Immune checkpoint inhibitors, VEGF/VEGFR inhibitors, TKIs, radiation → hypertension, endothelial injury, immune myocarditis; cytokine-mediated effects.
Time Course	Can be acute (during/soon after infusion), early (weeks–months), or late (months–years) depending on agent/dose.	Often subacute to early (weeks–months) but can occur acutely (e.g., ICI myocarditis) or late (vascular changes).
Biomarkers/Tests	Troponin, NT-pro-BNP, echo (LVEF/GLS), CMR (edema, LGE), strain imaging for subclinical dysfunction.	Troponin/BNP (if myocardial involvement), BP monitoring, endothelial function measures, CMR (myocarditis), angiography (vasospasm/microvascular).
Pathology/Imaging Signatures	Myocyte necrosis/apoptosis, mitochondrial swelling, interstitial fibrosis; CMR: LGE patterns consistent with myocarditis or cardiomyopathy.	Endothelial swelling, perivascular inflammation; CMR: edema and T2 changes (immune myocarditis) without primary myocyte toxin signature.
Risk Modifiers	Higher cumulative dose, prior CV disease, age, concomitant radiation, dual HER2 blockade, genetic susceptibility.	Pre-existing hypertension, autoimmune diseases, combination ICI therapy, high VEGF pathway inhibition, systemic inflammation.
Management Focus	Prevent injury/limit dose; early cardioprotective therapy (ACEi/ARB, β-blocker), schedule modifications, liposomal formulations; surveillance with echo/biomarkers.	Control mechanism: treat inflammation (e.g., steroids for ICI myocarditis), manage BP, address vasospasm/ischemia, optimize endothelial health; adjust or hold offending agent.
Prognosis Considerations	Persistent LV dysfunction risk if injury is extensive; recovery varies by agent and timing of intervention.	Often better prognosis if recognized early and mechanism addressed; recurrence risk depends on re-challenge and underlying biology.

The interplay between direct and indirect mechanisms complicates the clinical picture, as some drugs may exert both types of toxicity simultaneously. Radiation therapy is included because of the well-known added cardiotoxic effects alone or when used in combination with chemotherapy-most notably with anthracycline drugs [9].

Indirect *clinical* effects from changing treatment arise when cardiotoxicity necessitates dose reduction, interruption, or discontinuation of a specific cancer therapy. This can compromise oncologic efficacy, increase risk of cancer progression or recurrence, and require initiation of cardiovascular medications or interventions. There are significant cost implications as well. The management of cardiotoxicity often requires cessation of the offending agent and optimization of cardiovascular care, which may limit cancer treatment options [10]. This important area is discussed in greater detail in a subsequent section.

*When is Cardiotoxicity First Detected?*

For the top five classes of cancer drugs associated with cardiotoxicity, anthracyclines, HER2-targeted agents, immune checkpoint inhibitors, VEGF/TKIs, and proteasome inhibitors cardiotoxicity is typically first identified at different stages depending on the drug class and era of development (Table 3).

**Table 3:** Cancer Drug Classification, Cardiotoxicity Phenotypes, Potential Genotypes, and Time of Onset

Class	Individual Drugs (Examples)	Cardiotoxicity Phenotypes	Contributing Genotypes (if known)	Incidence (approximate)	Time from Initiation
Anthracyclines	Doxorubicin, Daunorubicin, Epirubicin	LV dysfunction, heart failure, arrhythmia, myocarditis	Variants in ABCB8, Top2β, p53, BAX	5–48% (dose-dependent)	Weeks to years
Alkylating Agents	Cyclophosphamide, Cisplatin, Ifosfamide	LV dysfunction, heart failure, arrhythmia, pericarditis	Unknown; possible metabolic variants	< 10% (varies by agent/dose)	Days to weeks
Taxanes	Paclitaxel, Docetaxel	Bradycardia, heart block, arrhythmia	Unknown	< 5%	Hours to days
Antimetabolites	5-Fluorouracil, Capecitabine	Coronary vasospasm, ACS, arrhythmia, sudden death	Unknown	1–10%	Hours to days
Endocrine Therapy	Tamoxifen, Anastrozole, Letrozole	Thromboembolism, atherosclerosis, arrhythmia	Unknown	< 5%	Weeks to months
HER2-Directed Therapies	Trastuzumab, Pertuzumab	LV dysfunction, heart failure	Unknown; possible ErbB2 variants	2–7%	Weeks to months
CDK4/6 Inhibitors	Palbociclib, Ribociclib	QTc prolongation	Unknown	< 5%	Weeks to months
VEGF Inhibitors/TKIs	Sunitinib, Sorafenib, Imatinib, Ponatinib	Hypertension, heart failure, ischemia, arrhythmia	Unknown	5–15%	Weeks to months
Immune Checkpoint Inhibitors	Pembrolizumab, Nivolumab, Ipilimumab	Myocarditis, arrhythmia, heart failure	HLA variants (suspected)	< 1% (myocarditis)	Days to months
Radiation Therapy	Chest/mediastinal radiation	CAD, cardiomyopathy, pericarditis, valvular disease	Unknown	Cumulative, up to 30% long-term	Years

LV: Left Ventricular, ROS: Reactive Oxygen Species, Top2β: Topoisomerase II beta, ACS: Acute Coronary Syndrome, TCA: Tricarboxylic Acid cycle, HER2:Human Epidermal Growth Factor Receptor 2, ErbB2: a gene encoding HER2 protein, CDK4/6: Cyclin-Dependent Kinase 4 and 6, QTc: Corrected QT interval, VEGF: Vascular Endothelial Growth Factor, TKIs: → Tyrosine Kinase Inhibitors, HLA: Human Leukocyte Antigen, CAD: Coronary Artery Disease

*How is Cardiotoxicity First Detected?*

The cardiotoxicity of a new cancer drug or regimen in 2025 is determined through a structured, multi-phase process guided by national regulatory agencies and public-private partnerships that integrates preclinical, clinical, and post-marketing evaluation, with additional safeguards for fast-track drugs intended for patients with standard treatment failure [11].

In the early investigation of cardiotoxicity associated with cancer therapeutics, an integrated approach should serve as the foundation for safe development and predictive human phenotyping. This includes elucidating cellular mechanisms of action, leveraging nuclear and tissue biomarkers, applying in silico modeling, and utilizing advanced single-cell or organoid systems.

Early-phase clinical trials (Phase I/II) include baseline and serial cardiac risk stratification and monitoring. The American College of Radiology recommends baseline assessment of ventricular function (e.g., left ventricular ejection fraction (LVEF), valvular function) and risk stratification using echocardiography or advanced imaging, such as cardiac Magnetic Resonance Imaging **(**MRI) before initiating therapy, even in asymptomatic patients. The American College of Cardiology (ACC), AHA, and Heart Failure Society of America (HFSA) recommend pretherapy evaluation and ongoing monitoring of cardiac function in patients receiving potentially cardiotoxic agents, with serial measurement of cardiac biomarkers (troponin, natriuretic peptides) considered reasonable for further risk stratification [12].

For fast-track drug approval, especially those for patients with standard treatment failure regulatory agencies may allow accelerated approval based on surrogate endpoints but require enhanced cardiac safety monitoring. This includes more frequent imaging (LVEF, global longitudinal strain), biomarker surveillance, and detailed adverse event reporting during trials and post-marketing surveillance. The HFSA and the ACC recommend risk-guided prevention strategies and multidisciplinary management for patients at high risk or with pre-existing cardiovascular disease [13].

Post-marketing surveillance is critical for detecting cardiotoxicity, with ongoing collection of real-world data on cardiovascular events, integration of advanced imaging, and biomarker algorithms to detect subclinical toxicity early. This comprehensive approach is essential to balance oncologic efficacy with cardiovascular safety in the era of rapid drug development and expedited approvals [14]. Adverse event detection rates and post-marketing safety surveillance have not kept pace with the acceleration in approval timelines [15].

## Impact of cardiotoxicity on treatment decisions and outcomes

Cardiotoxicity frequently results in treatment modifications. For example, in patients with ICI-myocarditis, immunotherapy is discontinued in most cases, with rechallenge only in highly selected cases given the high mortality risk [16]. For CAR T-cell therapy, severe cytokine release syndrome (CRS) may delay or prevent subsequent planned therapies, though the CAR T-cell infusion itself is a one-time treatment. For anthracyclines, a decline of left ventricular ejection fraction (LVEF) to < 50% or symptomatic heart failure typically mandates treatment discontinuation or dose reduction, potentially compromising cancer cure rates, particularly in curative settings like adjuvant breast cancer or lymphoma treatment [17].

The impact on cancer outcomes is substantial. Early discontinuation of effective cancer therapy due to cardiotoxicity may reduce response rates, compromise remission duration, and potentially affect overall survival. However, quantifying this impact is challenging as most studies focus on cardiac rather than oncologic endpoints. The cost implications are significant, including expenses for cardiac monitoring (serial echocardiography, biomarkers, cardiac MRI), cardiology consultations, cardioprotective medications, and management of heart failure when it develops [18].

For combination therapies, the increased cardiotoxicity risk must be weighed against superior anti-tumor efficacy. The four-fold increased risk of LVEF decline with v-Raf murine sarcoma viral oncogene homolog B1(BRAF) and Mitogen-Activated Protein Kinase Kinase (MEK) combinations necessitates more intensive cardiac surveillance, adding cost and potentially limiting treatment duration [19]. Similarly, the synergistic cardiotoxicity of anthracycline-trastuzumab combinations prompt a need for cardiac monitoring protocols and use of cardioprotective strategies, adding complexity and cost to cancer care [20].

Cancer treatment–associated cardiotoxicity significantly worsens patient outcomes and complicates clinical management by increasing both cardiac and cancer-related risks. This dual cardiac–oncologic toxicity necessitates integrated care approaches, including baseline cardiovascular risk stratification, serial biomarker and imaging surveillance, and in some instances a “permissive cardiotoxicity” strategy that balance continuation of cancer therapy with cardioprotective interventions. Ultimately, cardiotoxicity not only undermines long-term survival and quality of life [2] but also requires a multidimensional care framework involving oncology–cardiology collaboration to optimize both cardiac and cancer outcomes [21].

## Management of cardiotoxicity and surveillance

Management and surveillance of cardiotoxicity in cancer therapeutics have been addressed comprehensively by several major professional societies. The 2022 European Society of Cardiology (ESC) guideline, endorsed alongside the EHA, European Society for Radiotherapy and Oncology (ESTRO), and International Cardio-Oncology Society (IC-OS) offers a structured, multidisciplinary approach for cardiovascular care before, during, and after cancer treatment, emphasizing baseline risk assessment, prevention, monitoring, and management of cancer therapy–related cardiovascular toxicity (CTR-CVT). The American Society of Clinical Oncology (ASCO) expert consensus builds on that framework, recommending ACE inhibitors/ARBs, beta-blockers, and statins for cardio-protection during anthracycline and HER2 therapy, while also guiding echocardiography and biomarker schedules-especially troponin and ECG for ICIs-with rapid in-hospital management of ICI-associated myocarditis. The JACC: CardioOncology/ACC panel reinforces these surveillance principles, stressing that monitoring should be tailored based on class therapy, toxicity risk, and cost-effectiveness. They outline strategies for permissive cardiotoxicity and stress individualized decision-making in the absence of definitive outcome data. Finally, the NCCN Survivorship Guidelines highlight long-term screening for anthracycline-induced cardiomyopathy and cardiovascular disease risk, extending surveillance into survivorship care planning (Tables 4 and 5) [22] (www.nccn.org/guidelines; accessed March 14, 2026) [4, 13, 23].

**Table 4:** Guideline-Directed Therapy for Cardiotoxicity

Clinical Scenario	Recommendation	COR	LOE
**Established cancer therapy-related cardiomyopathy or HF**	Multidisciplinary discussion involving the patient about risk-benefit ratio of cancer therapy interruption, discontinuation, or continuation	1	B-NR
**Asymptomatic cancer therapy-related cardiomyopathy (EF < 50%)**	ARB, ACEi, and beta blockers to prevent progression to HF and improve cardiac function	2a	B-NR
**Patients with CV risk factors or known cardiac disease before potentially cardiotoxic therapy**	Pretherapy evaluation of cardiac function to establish baseline and guide choice of cancer therapy	2a	B-NR
**Patients with CV risk factors or known cardiac disease receiving potentially cardiotoxic therapy**	Monitoring of cardiac function for early identification of drug-induced cardiomyopathy	2a	B-NR
**Patients at risk of cancer therapy-related cardiomyopathy**	Initiation of beta blockers and ACEi/ARB for primary prevention (uncertain benefit)	2b	B-R
**Patients being considered for potentially cardiotoxic therapies**	Serial measurement of cardiac troponin for further risk stratification (might be reasonable)	2b	C-LD

**Table 5:** Baseline Assessment, Monitoring, and Surveillance

Cancer Therapeutic Class	Cardiotoxicity Profile	Baseline Assessment	During Treatment Monitoring	Post-Treatment Surveillance
**Anthracyclines** (doxorubicin, epirubicin, daunorubicin, idarubicin, mitoxantrone)	Cardiomyopathy/HF during or within 1 year; late effects relatively rare	LVEF + GLS (echo preferred, CMR second, MUGA third)	After cumulative dose > 250 mg/m² doxorubicin-equivalent, then every 50 mg/m²	LVEF at end of therapy, then 6–12 months post-completion
**HER2-targeted antibodies** (trastuzumab, pertuzumab, T-DM1, trastuzumab-deruxtecan)	Cardiomyopathy/HF during therapy; late effects relatively rare	LVEF + GLS (echo preferred)	Every 3 months (frequency varies by agent/duration)	As needed
**Proteasome inhibitors** (carfilzomib, bortezomib)	Cardiomyopathy/HF during therapy	LVEF + GLS (echo preferred)	If CV symptoms or HF evidence develops	As needed
**MEK inhibitors** (trametinib, selumetinib, cobimetinib, binimetinib)	Cardiomyopathy/HF during therapy	LVEF + GLS (echo preferred)	1 month after initiation, then every 2–3 months	As needed
**VEGF inhibitors** (sunitinib, pazopanib)	Cardiomyopathy/HF, hypertension, atherosclerotic disease during therapy	LVEF + GLS (echo preferred)	Cycle 1, possibly cycle 3; low yield beyond cycle 3 if asymptomatic	As needed
**Immune checkpoint inhibitors** (anti-PD-1, anti-PD-L1, anti-CTLA-4)	Myocarditis, cardiomyopathy/HF, arrhythmia typically early; late effects unknown	LVEF + GLS (echo preferred); consider baseline troponin/NT-proBNP	Serial biomarkers; imaging if symptoms/biomarker elevation	Immunomodulatory effects may persist after cessation

## Cardiovascular-oncology: An upstream approach to drug safety and optimal care

The FDA relies on internationally harmonized ICH guidelines rather than a standalone cardiotoxicity guidance for cancer therapeutics, but these requirements, especially ICH S9 (“Nonclinical Evaluation for Anticancer Pharmaceuticals”) explicitly mandate evaluation of cardiovascular risk using repeat-dose toxicity studies in two species with heart histopathology, electrocardiograms (ECGs), high-sensitivity troponin and other biomarkers, and incorporation of safety pharmacology endpoints from ICH S7A/B and hERG/TQT testing under ICH E14/S7B (ICH – International Council for Harmonisation of Technical Requirements for Pharmaceuticals for Human Use; S7A – Safety Pharmacology Studies for Human Pharmaceuticals general safety pharmacology guideline); S7B – Nonclinical Evaluation of the Potential for Delayed Ventricular Repolarization (QT Interval Prolongation) by Human Pharmaceuticals; E14 – Clinical Evaluation of QT/QTc Interval Prolongation and Proarrhythmic Potential for Non-Antiarrhythmic Drugs; hERG – human Ether-à-go-go-Related Gene (encodes the cardiac potassium channel I_Kr, critical for repolarization); TQT – Thorough QT/QTc Study to assess arrhythmic potential); these nonclinical and clinical cardiac safety assessments are routinely followed by sponsors and fully required to support both investigational new drug (IND) and new drug application (NDA) submissions (www.fda.gov/about-fda/oncology-center-excellence/oncology-center-excellence-guidance-documents; accessed March 14, 2026).

Minimizing harm from cancer therapeutics requires a dual approach that integrates optimal drug design with proactive strategies to prevent or mitigate cardiovascular toxicity. This begins with incorporating cardiotoxicity risk assessment early in the drug development pipeline using predictive biomarkers, in silico modeling, and preclinical systems that simulate cardiac stress under oncologic treatment conditions. Concurrently, designing therapies with improved selectivity and reduced off-target effects, such as targeted delivery systems or molecular modifications can lower cardiac burden [3, 24, 25].

To best capture cardiotoxicity signals during cancer drug development, a clinical trial should integrate cardiovascular safety objectives alongside efficacy endpoints, using a composite cardiovascular (CV) outcome that includes major adverse cardiac events (MACE+), clinically significant arrhythmias, QTc prolongation, and imaging-based measures such as LVEF and global longitudinal strain (GLS) [26, 27]. The design should incorporate baseline and serial assessments of cardiac biomarkers (e.g., high-sensitivity troponin, NT-proBNP), echocardiography, ECG monitoring, and optional cardiac MRI for agents with potential myocarditis or fibrosis risk. Stratification by CV risk tiers and extended follow-up after treatment (12–24 months) might increase detection of both early and late toxicity [28]. Exposure–response analyses, digital health sub-studies, and linkage to real-world data can provide mechanistic insights and long-term surveillance, making cardiotoxicity detection robust and clinically meaningful [29]. *This will be discussed in detail in part 3 of this series.*

In parallel, developing and co-administering cardioprotective agents, such as beta-blockers, ACE inhibitors, or novel cardioprotective compounds, alongside cancer drugs can help preserve cardiac function and either prevent or attenuate other cardiovascular risk. Finally, adaptive clinical trial designs that monitor cardiovascular endpoints in real time enable early intervention and personalized risk mitigation, ensuring therapeutic efficacy without compromising long-term cardiovascular health.

Cardio-protective agents would under ideal circumstances be developed concomitantly with cancer treatments, targeting multiple mechanisms of cardiovascular toxicity including oxidative stress, mitochondrial dysfunction, neurohormonal activation, and inflammation. Current evidence supports several therapeutic targets, though optimal strategies remain under investigation.

## Theoretical targets and potential drugs

Neurohormonal blockade represents the most extensively studied approach. Angiotensin converting-enzyme (ACE) inhibitors, angiotensin receptor blockers (ARBs), and beta-blockers target the renin-angiotensin-aldosterone system and sympathetic activation, which contribute to cardiomyocyte stress and remodeling [30]. Carvedilol offers additional antioxidant benefits beyond beta-blockade. Sacubitril/valsartan (ARNI) has shown promise in preserving LVEF and global longitudinal strain in the SARAH trial, though hypotension remains a concern [30, 31].

Metabolic modulation through SGLT2 inhibitors (dapagliflozin, empagliflozin) represents an emerging frontier. These agents provide cardio-protection through metabolic optimization, anti-inflammatory effects, and reduced oxidative stress [32].

Dexrazoxane, an iron chelator, reduces anthracycline-induced oxidative stress and DNA damage through Top2β inhibition [33]. It remains the only FDA-approved cardioprotective agent for anthracycline therapy, recommended for high-risk patients.

Novel combination approaches are under investigation. Preclinical evidence suggests GLP-1 receptor agonists (semaglutide) combined with PCSK9 inhibitors (inclisiran) may provide synergistic cardio-protection by reducing oxidative stress, ferroptosis, and inflammatory signaling (NLRP3, MyD88, NF-κB pathways) while potentially enhancing chemosensitivity [34].

## Clinical trial design considerations

Patient selection should employ risk stratification, incorporating both traditional cardiovascular risk factors and cancer therapy-specific risks (e.g., cumulative anthracycline dose, radiation field/dose, HER2-targeted therapy). The Heart Failure Association–International Cardio-Oncology Society (HFA-ICOS) risk tool is a clinical risk-assessment instrument designed to stratify cancer patients by their risk of cardiotoxicity prior to starting cancer treatment [35]. It could potentially be employed as a proactive risk-stratification framework rather than a predictive model. By applying HFA-ICOS at baseline, investigators could categorize participants into cardiovascular risk tiers (low, moderate, high, very high) based on comorbidities, prior exposures, and cardiac function. This, in turn could enable tier-specific surveillance intensity-such as more frequent biomarkers and imaging for high-risk patients while generating structured data to detect early cardiac signals. Integrating this approach into trial protocols would enhance systematic monitoring and create a standardized dataset for regulatory review, even when a newly developed drug’s cardiac risk is uncertain. The key elements would be including cardiovascular endpoints in the early stages of drug development to include baseline and serial electrocardiograms, echocardiogram with GLS, hs-troponin, NT-proBNP, and clinical endpoints (Tables 6 and 7).

**Table 6:** Cardiovascular Endpoints by Trial Phase

Phase	Endpoint	Assessment Method	Monitoring Frequency
**Phase 1**	Subclinical LV dysfunction (GLS decline > 15%)	Echocardiogram with GLS	Baseline; every 8–12 weeks (high-risk: every 6 weeks)
	Biomarker elevation (hs-Troponin, NT-proBNP)	Central lab assays	Baseline; each cycle; 24–72 h post-dose for first 2–3 cycles (high-risk)
	QTc prolongation	12-lead ECG	Baseline; pre-dose each cycle
	Arrhythmias	ECG + telemetry (if inpatient)	Continuous during infusion; ECG each cycle
	Early CTRCD composite	Echo + biomarkers	Same as above
**Phase 2**	CTRCD (ESC definition: LVEF drop ≥ 10% to < 50%)	Echocardiogram	Baseline; every 8–12 weeks
	GLS decline	Echocardiogram with strain	Baseline; every 8–12 weeks
	Biomarker trends	hs-Troponin, NT-proBNP	Baseline; each cycle
	Clinical HF events	Clinical exam + CTCAE grading	Ongoing
	Arrhythmias	ECG	Each cycle
	Hypertension	BP measurement	Each visit
**Phase 3**	CTRCD (primary endpoint)	Echocardiogram	Baseline; every 12 weeks; end of treatment
	HF hospitalization	Clinical adjudication	Continuous
	Myocarditis	Cardiac MRI if suspected	As clinically indicated
	MACE (CV death, MI, stroke)	Adjudication committee	Continuous
	Composite endpoint (CTRCD + HF + CV death)	Echo + clinical + adjudication	Continuous

**Abbreviations: CTRCD**: Cancer Therapy–Related Cardiac Dysfunction; **GLS**: Global Longitudinal Strain; **LVEF**: Left Ventricular Ejection Fraction; **hs-Troponin**: High-sensitivity Troponin; **NT-proBNP**: N-terminal pro-B-type Natriuretic Peptide; **ECG**: Electrocardiogram; **HF**: Heart Failure; **CTCAE**: Common Terminology Criteria for Adverse Events; **BP**: Blood Pressure; **MRI**: Magnetic Resonance Imaging; **MACE**: Major Adverse Cardiovascular Events; **MI**: Myocardial Infarction

**Table 7:** Exploratory Biomarkers for Early Cardiotoxicity Detection

Category	Biomarker	Sample Type	Detection Method	Suggested Time Points
**Injury**	hs-Troponin I/T	Plasma/Serum	Immunoassay (ELISA)	Baseline; each cycle; 24–72 h post-dose (Phase 1); q8–12 weeks (Phase 2–3)
	H-FABP	Plasma	ELISA	Same as above
	CK-MB	Plasma	Immunoassay	Same as above
**Stress/Remodeling**	NT-proBNP / BNP	Plasma	Immunoassay	Baseline; each cycle; q8–12 weeks
	ST2	Plasma	ELISA	Baseline; q8–12 weeks
	Galectin-3	Plasma	ELISA	Baseline; q8–12 weeks
**Inflammation**	IL-6, IL-1β, IL-18	Plasma	Multiplex ELISA / Luminex	Baseline; each cycle; at suspected toxicity
	TNF-α	Plasma	ELISA	Same as above
	CRP	Plasma	Immunoassay	Baseline; each cycle
**Oxidative Stress**	Myeloperoxidase (MPO)	Plasma	ELISA	Baseline; q8–12 weeks
	Caspase-cleaved fragments	Plasma	ELISA	Baseline; q8–12 weeks
**Molecular**	cf-DNA	Plasma	qPCR / Digital PCR	Baseline; each cycle; at suspected toxicity
	MicroRNAs (miR-1, miR-133a, miR-208a)	Plasma	qRT-PCR	Baseline; each cycle
	Extracellular Vesicles (EVs)	Plasma	Nanoparticle tracking + LC-MS	Baseline; exploratory
	GDF-15	Plasma	ELISA	Baseline; q8–12 weeks

**Abbreviations: ELISA**: Enzyme-Linked Immunosorbent Assay; **qPCR**: Quantitative Polymerase Chain Reaction; **LC-MS**: Liquid Chromatography–Mass Spectrometry; **cf-DNA**: Circulating Cell-Free DNA; **NT-proBNP**: N-terminal pro-B-type Natriuretic Peptide; **hs-Troponin**: High-sensitivity Troponin; **H-FABP**: Heart-type Fatty Acid Binding Protein

Key challenges include the relatively low overall event rates in contemporary cancer therapy -at least in early stages of development, heterogeneity in cancer types and treatment regimens, and the need to demonstrate non-interference with oncologic efficacy. Multidisciplinary collaboration between oncologists, cardiologists, clinical trialists, the pharmaceutical industry, and regulatory bodies is essential, with personalized approaches considering individual patient factors, cancer stage, drug mechanism(s) of action, and cardio-protective therapy options, including the inclusion of an experimental arm of a phase 2B or 3 clinical trial if cardiotoxicity signals were detected in phase 1–2 A studies.

## Conclusion

Cardiotoxicity remains a critical challenge in contemporary cancer therapeutics, influencing treatment decisions, patient outcomes, and healthcare costs. Its multifactorial nature, encompassing direct myocardial injury, indirect systemic effects, and complex drug interactions underscores the need for proactive strategies that integrate cardiovascular safety into every stage of drug development. Early detection through biomarkers and imaging, risk stratification, and multidisciplinary management are essential to mitigate harm while preserving oncologic efficacy. Future directions must prioritize adaptive trial designs, incorporation of cardio-protective agents, and real-world surveillance to ensure that therapeutic innovation does not come at the expense of cardiovascular health. Ultimately, a collaborative Cardio-Oncology framework is indispensable for optimizing both cancer and cardiac outcomes.
